# If You Go Up, You May Fall: A Rare Case of Rapidly Progressing Multifocal High-Altitude Vascular Thrombosis

**DOI:** 10.7759/cureus.9860

**Published:** 2020-08-19

**Authors:** Divya Pramod Nimmatoori, Namrata Singhania, Saurabh Bansal, Anil Singh, Subhankar Samal

**Affiliations:** 1 Internal Medicine, GreenField Health, Portland, USA; 2 Hospital Medicine, Mount Carmel Hospital, Columbus, USA; 3 Internal Medicine, University of Illinois, Peoria, USA; 4 Hospital Medicine, Geisinger Medical Center, Scranton, USA; 5 Internal Medicine, Ascension Health, Milwaukee, USA

**Keywords:** high altitude, thromboembolism, mesenteric thrombosis

## Abstract

A high-altitude (HA) exposure can lead to hypercoagulability, increasing the risk for major thromboembolic events. The spontaneous vascular thrombosis can be arterial or venous, and may occur after a short or extended stay at higher elevations. Diagnosis is often challenging, and delays in treatment can lead to inevitable and disabling sequelae. With a limited scope of experimental studies and trials to prove HA thrombogenicity, a greater awareness of the diversity of presentations and management is imperative to those engaged in treating such patients. We report a case of rapidly progressing HA vascular thrombosis.

## Introduction

A high-altitude (HA) exposure during air travel, mountaineering, or sports activities is thought to create an enhanced prothrombotic milieu, thus predisposing to serious thromboembolic events. This phenomenon is thought to have a multifactorial trait, both genetic and acquired, caused by a complex interplay of various elements. Virchow’s triad, including venous stasis, hypercoagulability, and endothelial injury, appears to be present at HA and extreme environmental conditions leading to hypoxemia supporting the occurrence of thromboembolism [1]. Herein, we report a rare case of rapidly progressing HA vascular thrombosis with massive pulmonary thromboembolism and superior mesenteric artery thrombosis treated with thrombolytics and surgery, respectively.

## Case presentation

A 61-year-old male patient with a past medical history of essential hypertension presented to the hospital with complaints of sudden onset of shortness of breath for three days, and abdominal pain, nausea, vomiting, and loose stools for two days. He reported traveling to Sikkim (an HA state in India nestling at an approximate altitude of 9,000 feet in Himalayan mountains) for a family vacation five days prior to hospitalization. His symptoms developed two days after his return. The breathlessness was associated with palpitations, profuse sweating, and a stabbing pleuritic chest pain. This was followed by a sudden onset of diffuse abdomen pain. The pain was aggravated by food and was associated with multiple episodes of loose stools and non-projectile non-bilious vomiting. The patient denied having a cough, fever, hemoptysis, orthopnea, paroxysmal nocturnal dyspnea, history of syncopal attacks, recent trauma, immobilization, and black tarry or bloody stools. He denied any history of thrombotic symptoms in the past. There was no family history of hypercoagulability. He was an active smoker. His temperature was 98.6^o^F, pulse was 120 beats per minute, respiratory rate was 32 per minute, blood pressure was 88/70 mmHg, and SpO_2_ was 85% on room air. Lung examination revealed normal vesicular breath sounds and no adventitious sounds. Heart sounds were normal with no murmurs. An elevated jugular venous pressure was noted. Abdominal examination showed mild diffuse tenderness.

**Figure 1:**
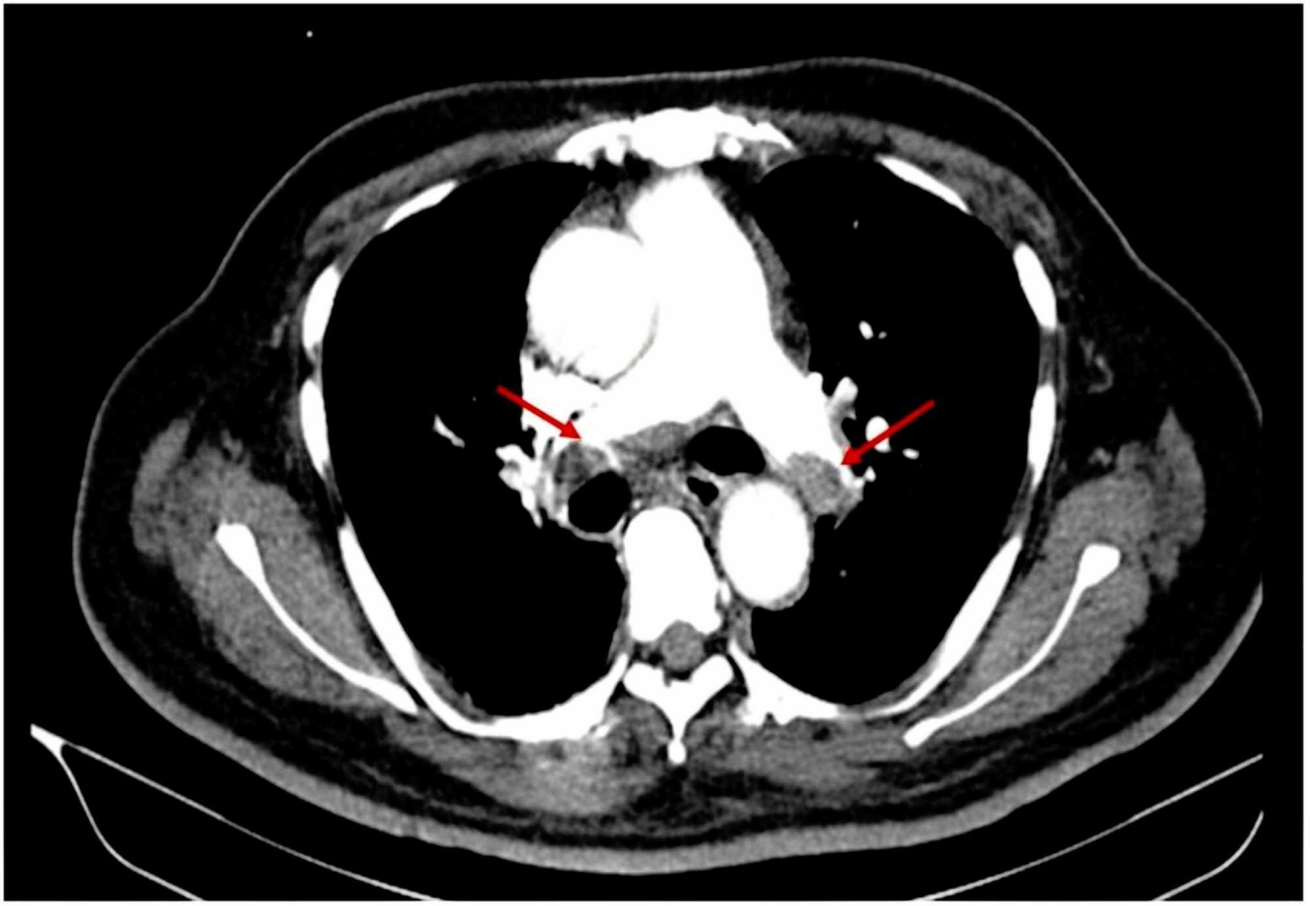
Bilateral Pulmonary Embolism (PE) CT angiography of the chest showing large filling defects in bilateral pulmonary arteries, segmental, and subsegmental arteries, suggesting massive PE.

Laboratory investigations showed normal serum electrolytes, liver function tests, and lipid profile. His hemoglobin was 18 g/dL (normal range [NR]: 12.0-15.3 g/dL), hematocrit was 66% (NR: 36.0%-45.2%), white blood cell count was 10.2 K/mm^3 ^(NR: 4.00-10.80 K/mm^3^), platelet count was 320 K/mm^3 ^(NR: 140-400 K/mm^3^), serum creatinine was 0.9 mg/dL (NR: 0.5-1.0 mg/dL), blood urea nitrogen was 22 mg/dL (NR: 6-20 mg/dL), prothrombin time was 14 seconds (NR: 11.0-12.5 seconds), activated partial thromboplastin time was 22 seconds (NR: 30.0-40.0 seconds), and international normalization ratio was 1.5 (NR: <1.1). Factor V Leiden, antiphospholipid antibody test, and protein C and S tests were normal. Chest radiograph showed a dilated descending pulmonary artery. Electrocardiogram showed sinus tachycardia with no significant ST-T wave changes. Troponin was negative, and D-dimer was 6,000 ng/mL. CT angiography of the chest with intravenous contrast showed large filling defects in bilateral pulmonary arteries, segmental, and subsegmental arteries, suggestive of an acute massive pulmonary thromboembolism (Figure 1).

**Figure 2:**
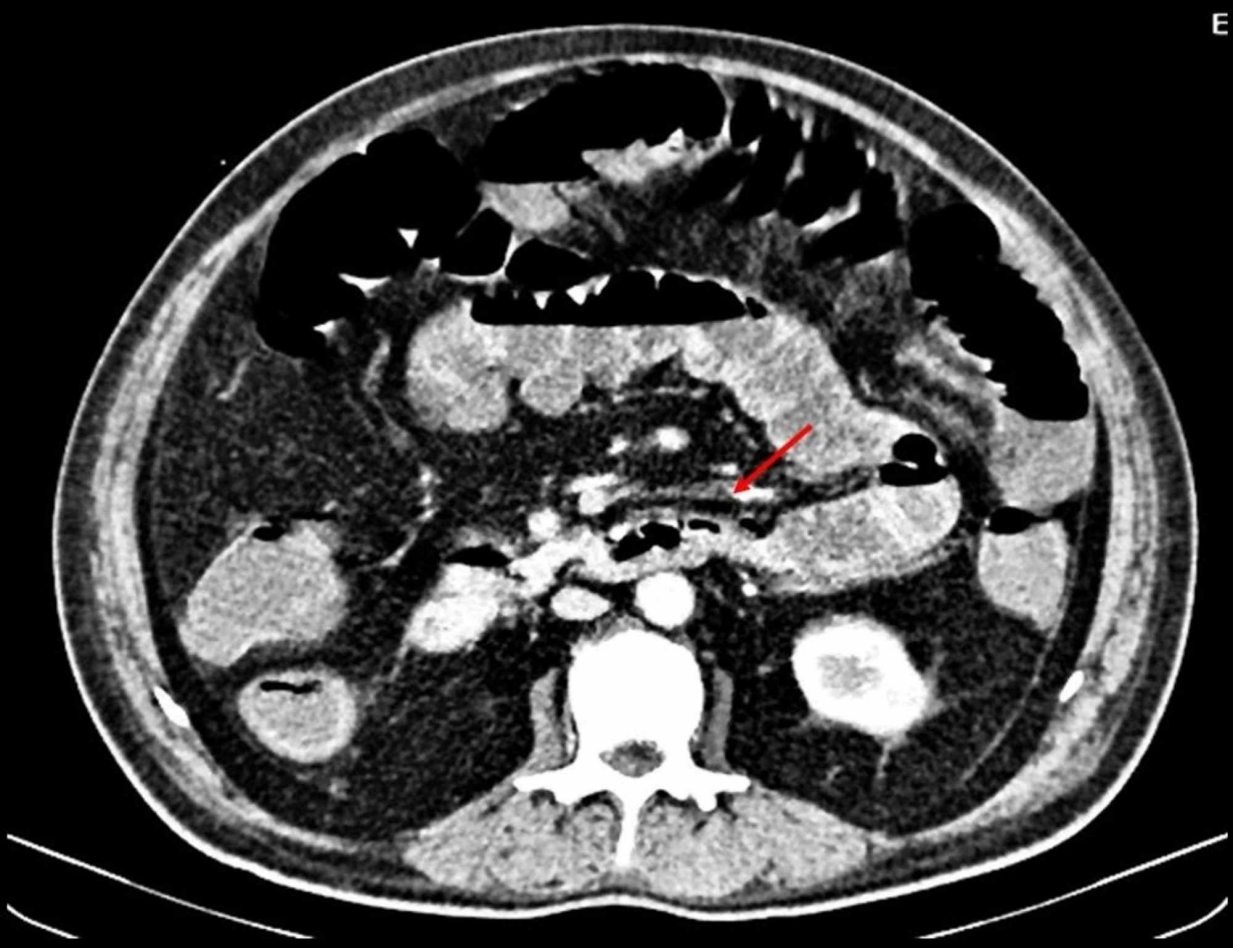
Superior mesenteric artery (SMA) thrombosis Contrast-enhanced CT of the abdomen showing an intraluminal filling defect in SMA indicating SMA thrombosis.

Transthoracic echocardiogram showed right atrial and right ventricular dilation, and left ventricular ejection fraction of 50% suggesting impending right ventricular failure. Systemic thrombolytic therapy using recombinant human non-glycosylated tissue plasminogen activator (100 mg administered over two hours) was administered followed by anticoagulation with unfractionated heparin (5,000 international units four times a day, 24 hours later). His dyspnea improved afterwards, but continued to have severe abdominal pain. Contrast-enhanced CT of the abdomen was performed that showed pneumatosis intestinalis with an intraluminal filling defect in superior mesenteric artery, suggestive of thrombosis (Figure 2).

**Figure 3:**
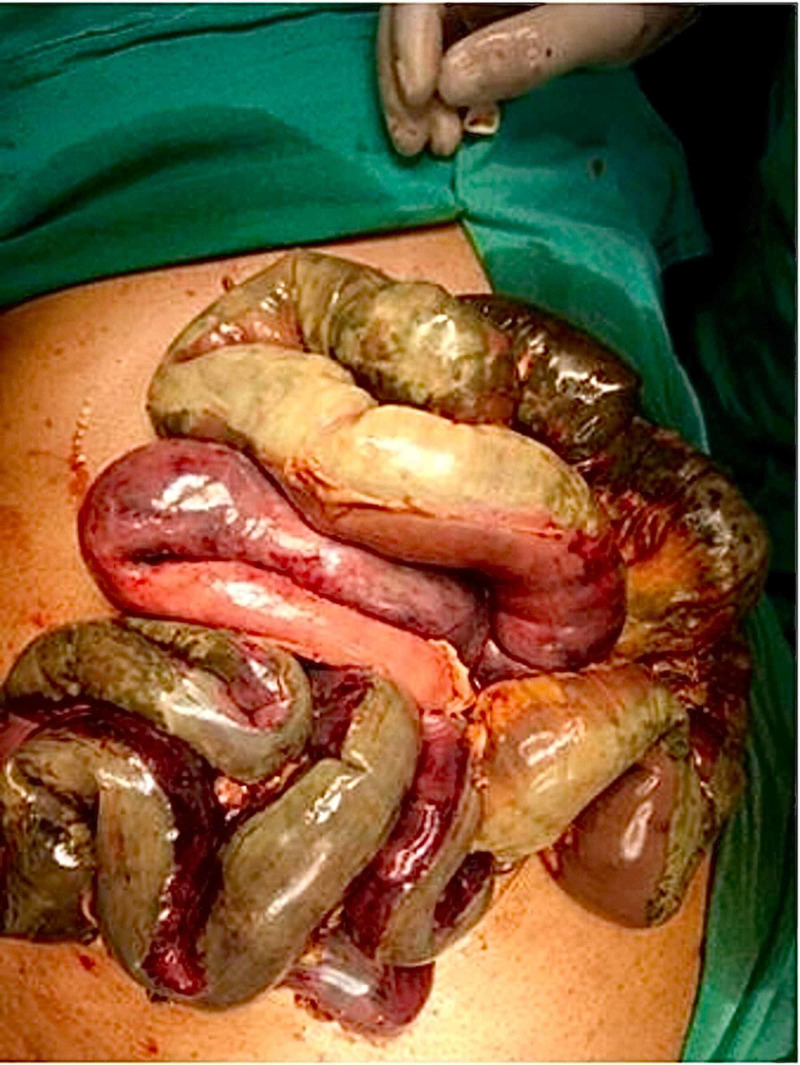
Explorative laparotomy revealing a dusky small bowel from the duodenal-jejunal flexure to the ileocecal valve.

As he was hemodynamically unstable, an explorative laparotomy was performed, which showed dusky small intestine suggestive of mesenteric thrombosis (Figure 3).

A jejunocolic anastomosis was performed, and broad-spectrum antibiotics were started. Even after the surgery, he continued to decline and developed multiorgan failure with acute hypoxemic respiratory failure requiring invasive ventilation, and severe acute kidney injury requiring continuous renal replacement therapy. He also suffered left lower limb ischemia and eventually succumbed despite the best efforts.

## Discussion

HA illness (HAI) includes a range of pathologic syndromes that develop following an initial ascent to HA or following a further ascent while already at HA. HAI includes acute mountain sickness, HA cerebral edema, and HA pulmonary edema. Acute mountain sickness usually has a milder presentation and faster resolution of symptoms and in severe cases leads to cerebral edema [2]. The incidence of HA pulmonary edema in climbers of the Himalayas and the Alps with an ascending rate >600 meters per day is around 4% [3,4]. HAI is usually associated with rapid ascent to heights over 9,000 feet above the mean sea level.

The factors associated with high risk for HAI include a prior history of HAI, the rate of ascent, level of elevation attained, vigorous exertion prior to acclimatization, use of alcohol, and presence of baseline neuromuscular disease or pulmonary hypertension [5]. Other important variables that determine an individual’s predisposition to HAI are genetic susceptibility and the extent of hypoxemic stress. It is presumed that the hypoxemia itself alters the coagulation cascade which creates a prothrombotic environment. An uncommon and serious peril of HA travel is vascular thrombosis which is usually associated with risk factors, such as prolonged exposure to extreme altitudes along with cold weather, dehydration, hemoconcentration, constrictive clothing, and enforced stasis [1]. Polycythemia is an independent risk factor for thrombosis that was seen in our patient [6]. The pulmonary hypertension of HA is also related to intravascular activation of the coagulation pathway. The lengthier stays at HA with extreme weather are associated with a 30 times higher risk of spontaneous vascular thrombosis, with venous thrombosis being more common than arterial thrombosis. Mesenteric or portosystemic thrombosis, involving the superior mesenteric artery, is rare and challenging to diagnose. This can lead to infarction of the gut. The presentation varies from a poorly localized abdominal pain with distension and fever to vomiting and gastrointestinal bleeding mimicking other diseases like pancreatitis, perforated peptic ulcer, and peritonitis. Classic presentation is pain out of proportion to physical exam. Angioplasty is indicated in impending bowel ischemia, and exploratory laparotomy is indicated in patients with frank bowel ischemia [6]. 

Gradual ascent is the safest method to prevent or decrease the risk of HAI. Generally, individuals who normally reside below 5,000 feet elevation should avoid an abrupt ascent to sleeping altitudes above 9,200 feet. Sedative hypnotics should be avoided during acclimatization. Abstinence from alcohol should be considered. Vigorous exertion at HA should be avoided. It is advised to bring the individuals down to lower more comfortable altitude if they start developing symptoms at HA.

## Conclusions

This case report supports the prothrombotic nature of HA and identifies serious concerns about the impact of HA exposure on vascular health. Pathogenesis is likely related to hypoxemia at HA. Prior history of HAI and rate of ascent are important risk factors. Venous thromboembolism is more commonly seen than arterial. Diagnosis can be challenging, and delays in appropriate treatment can lead to serious outcomes such as death.
